# The effects of the HEALTHY study intervention on middle school student dietary intakes

**DOI:** 10.1186/1479-5868-8-7

**Published:** 2011-02-04

**Authors:** Anna Maria Siega-Riz, Laurie El Ghormli, Connie Mobley, Bonnie Gillis, Diane Stadler, Jill Hartstein, Stella L Volpe, Amy Virus, Jessica Bridgman

**Affiliations:** 1 Carolina Population Center, University of North Carolina at Chapel Hill. NC. USA; 2 Biostatistics Center, George Washington University, WA, USA; 3 University of Nevada Las Vegas, NV, USA; 4 Health Promotion Department, University of Pittsburgh Medical Center Health Plan, PA, USA; 5 Division of Health Promotion & Sports Medicine, Oregon Health & Science University, OR, USA; 6 University of California, Irvine, CA, USA; 7 Division of Biobehavioral and Health Sciences, School of Nursing, University of Pennsylvania, PA, USA; 8 Center for Obesity Research & Education Temple University, PA, USA; 9 School of Nursing, University of North Carolina at Chapel Hill. NC, USA

## Abstract

**Background:**

The HEALTHY study was designed to respond to the alarming trends in increasing rates of overweight, obesity, and type 2 diabetes mellitus in youth. The objective of this analysis was to examine the effects of the HEALTHY study on student self-reported dietary intakes (energy, macronutrients and grams consumed of selected food groups).

**Methods:**

HEALTHY was a cluster-randomized study in 42 public middle schools. Students, n = 3908, self-reported dietary intake using the Block Kids Questionnaire. General linear mixed models were used to analyze differences in dietary intake at the end of the study between intervention and control schools.

**Results:**

The reported average daily fruit consumption was 10% higher at the end of the study in the intervention schools than in the control schools (138 g or approximately 2 servings versus 122 g, respectively, p = 0.0016). The reported water intake was approximately 2 fluid ounces higher in the intervention schools than in the control (483 g versus 429 g respectively; p = 0.008). There were no significant differences between intervention and control for mean intakes of energy, macronutrients, fiber, grains, vegetables, legumes, sweets, sweetened beverages, and higher- or lower-fat milk consumption.

**Conclusion:**

The HEALTHY study, a five-semester middle school-based intervention program that integrated multiple components in nutrition, physical education, behavior change, and social marketing-based communications, resulted in significant changes to student's reported fruit and water intake. Subsequent interventions need to go beyond the school environment to change diet behaviors that may affect weight status of children.

**Clinical Trials Registration:**

NCT00458029

## Introduction

Rates of overweight, obesity, and type 2 diabetes mellitus in youth have increased dramatically during the last three decades[1-3]. The HEALTHY study was designed to respond to these alarming trends. HEALTHY was a randomized, multicenter, middle school-based primary prevention trial designed to moderate risk factors for type 2 diabetes mellitus[4]. Modifiable risk factors measured were indicators of adiposity and glycemic dysregulation: body mass index (BMI), fasting glucose concentrations, and fasting insulin concentrations. The intervention program integrated multiple components in four areas: nutrition, physical education, behavior change, and social marketing-based communications[4]. Significant differences at end of study between intervention and control schools were found in mean BMI z score, the percentage of children who were ≥ 95^th ^percentile of BMI, the percentage of children with waist circumference >90 cm, and mean fasting insulin levels. However, no significant differences were found in the percentage of children ≥ 85^th ^percentile of BMI, with fasting insulin ≥ 30 μU/dL, with fasting glucose ≥ 100 mg/dL, in mean fasting glucose levels, or in mean waist circumference[5].

The HEALTHY nutrition intervention component was designed to implement changes in the quantity and nutritional quality of food and beverages available to students throughout the total school food environment, with an emphasis on changes likely to reduce the risk of overweight, obesity, and type 2 diabetes[6]. The total school food environment included cafeteria meals and after-school snacks provided through federal meal programs (the School Breakfast Program, National School Lunch Program, After-School Snack Program, and Supper Program), as well as a la carte venues, such as snack bars and school stores, vending machines, fundraisers, and classroom parties and celebrations. The intervention goals and strategies targeted: 1) high-fat foods, 2) fruits and vegetables, 3) high-fat/calorie snacks and desserts, 4) added-sugar beverages, and 5) fiber-rich foods including grain-based foods and legumes (see Table 1). The intervention also aimed to improve student dietary intake outside of the school environment through messages about healthy eating, cafeteria-based educational events, taste tests to introduce new food items, and nutrition education provided in the classroom and through parent newsletters.

**Table 1:** The Nutrition Intervention Goals and Corresponding Strategies of the Healthy Study

Goals	Strategies
1. Lower the average fat content of food served in schools.	Offer French fry type vegetable or flash-fried potato products in serving size of ≤200 calories or meal equivalent portion only once per week, either at breakfast or lunch. Offer only reduced fat and/or baked chips with ≤200 calories per package or serving. Offer only ≤1% fat milk. Replace the highest fat bread products with lower fat options and/or reduce the frequency offered/served. Replace the highest fat entrees with lower fat options and/or reduce the frequency offered/served.
2. Serve at least 2 servings of fruit and/or vegetables per student on NSLP and at least 1 serving per student on SBP each day.	Offer at least three different fruits and/or vegetables at NSLP every day. Offer at least two different fruits and/or vegetables at SBP every day.
3. Serve all dessert and snack foods with ≤200 calories per single serving size and or package.	Modify dessert and snack food offerings to ≤200 calories per single serving size package and/or serving, excluding nuts and seeds (This strategy does not apply to after-school snacks).
4. Eliminate milk greater than 1% fat, all other added sugar beverages, and 100% fruit juice (100% fruit juice may only be served as ≤ 6 ounces as part of SBP and/or after-school snacks).	Offer only ≤1% fat milk. Eliminate all added sugar beverages (with the exception of flavored 1% milk and nonfat milk). Eliminate 100% fruit juice from vending, a la carte, school stores, and NSLP, and limit 100% fruit juice at SBP to ≤ 6 ounces.
5. Serve at least 2 servings of grain-based foods and/or legumes (≥2 grams of fiber per serving) per student on NSLP and at least 1 serving per student on SBP each day.	Offer at least three different high fiber grain-based foods and/or legumes (≥2 g fiber/serving) at NSLP every day. Offer at least two different high fiber grain-based foods and/or legumes (≥2 g fiber/serving) at SBP every day.

Abbreviations: NSLP, National School Lunch Program; SBP, School Breakfast Program

This paper presents data on student self-reported dietary intakes (energy, macronutrients and grams consumed of selected food groups) at baseline and at the end of the study and tests for difference between the two groups as a result of the three-year long intervention. A sub analysis for children in the obese category is presented because preliminary analysis indicated that the intervention worked better in this subpopulation.

## Methods

HEALTHY was a cluster-randomized study in 42 public middle schools. Seven centers participated (Baylor College of Medicine, Houston, TX; Oregon Health & Science University, Portland, OR; University of California at Irvine, Irvine, CA; Temple University, Philadelphia, PA; University of North Carolina at Chapel Hill, Chapel Hill, NC; University of Pittsburgh, Pittsburgh, PA; and the University of Texas Health Science Center at San Antonio, San Antonio, TX), with six schools (three intervention and three control) at each center. For inclusion in the study, each school's student population was required to be at least 50% minority (African American, Hispanic/Latino and/or American Indian) and/or greater than 50% eligible for Federal reimbursable meals at free or reduced-cost.

The study followed students from sixth (ages 10-11) through eighth grade (ages 13-14), starting in the fall of 2006. Protocols for data collection and a detailed description of the intervention have been previously published[4,7]. All intervention components were delivered over five semesters (second semester of 6^th ^grade, both semesters of 7^th ^grade, and both semesters of 8^th ^grade), and each semester's activities focused on a specific theme: consuming water versus sweetened beverages; increasing physical activity and reducing sedentary behavior; consuming high quality versus low quality foods; understanding energy balance; and strength, balance and making choices for life.

Data collection included measurements made at the school, grade and student levels. At the student level this included sociodemographics, anthropometrics, blood pressure, fasting blood draw for glucose, insulin, lipids, and HbA1c, sexual maturity, quality of life, dietary intake, physical activity/inactivity, and fitness level. Written parental consent and child assent were required for participation in data collection. The study was approved by each participating university's Institutional Review Board.

### Dietary Intakes

Students self-reported their dietary intake using the Block Kids Questionnaire, a semi-quantified food frequency questionnaire (FFQ) that asks about consumption of approximately 100 food items during the past week; portion sizes are elicited using a serving size visual[8]. The questionnaire was administered by trained study staff to small groups of consented students, with the questionnaire and a serving size visual provided in both English and Spanish. Staff members were available to clarify any questions posed by the students, and students were encouraged to answer all questions. The first few questions were read aloud and explained, if necessary, by a trained interviewer until it was determined that participants understood the question and the completion of the task. The location and size of the groups were determined by the individual centers, depending on what was possible at each school given class schedules and other constraints.

Five questions pertinent to the intervention goals were added to the original questionnaire to elicit the intake of: 1) whole grain cereals; 2) regular versus low fat salad dressing; 3) whole grain pasta, couscous, and brown rice; 4) regular versus baked chips; and 5) water intake. The original questionnaire was validated in numerous studies and in a variety of populations[9-12]. It was redesigned for use with children by Nutrition Quest, formerly Block Dietary Data Systems. Two studies have been conducted that compare this tool to other self-reported food intake assessment methods. One study by Block et al. was conducted among 74 8-to-10-year-old African-American children attending school in Philadelphia; the comparison tool was one 24-hour recall. Correlation coefficients ranged from 0.40 to 0.50 for total energy, fat, saturated and monounsaturated fat, carbohydrate, fiber and calcium (unpublished Abstract presented at the 4^th ^International Conference on Dietary Assessment Methods). Recently, Cullen et al. (2008) compared the Block Kids Questionnaire to two 24-hour recalls in 83 children from age 10 to 17 years[13]. Correlations for nutrients ranged from 0.29 to 0.69 and for food groups from -0.03 to 0.74. Stratification of the data by age group illustrated that correlations for the nutrients, but not food groups, were higher among the older adolescents[13].

Dietsys+Plus version 5.6 was used to analyze the questionnaires[14]. This software produces estimates of usual intake for 46 nutrients and calculates daily frequency, daily gram amounts for each individual food item and up to 20 food groups that can be modified by the investigator. The food composition table for Dietsys has been updated with nutrient values based on data from national nutrition monitoring studies[15]. Using the output from this software, several food items were aggregated to fit the definition of food groups in the HEALTHY study's nutrition intervention goals. For example, all vegetables were included in the vegetable group with the exception of French fries; pinto beans and refried beans were included in the legumes group; ice cream, frozen yogurt, cookies, donuts, cakes, pie, fruit crisp, cobbler, candies, and sweetened cereals were included in the sweets group; and sweetened beverages included fruit drinks, sport drinks, energy drinks, etc.; 100% fruit juice and flavored milks were not included.

## Statistical Analysis

A total of 4603 students were included in the HEALTHY study cohort (measured at both baseline and the end of the study). Of those, 4560 (99%) completed the Modified Block Kids FFQ at both time points. Students who reported <500 or >5000 calories per day (implausible intakes as previously defined in the literature)[16] were excluded, resulting in a sample size of 3908 students (85% of the study cohort) available for this analysis.

Descriptive statistics (mean or geometric mean, standard deviation, median, and inter-quartile range or percent) were calculated for the student characteristics of interest as well as for the dietary variables for all children with useable data. Most of the macronutrient (carbohydrate, protein, and fat) and energy data best fit a log-normal distribution. Therefore, the natural log nutrient intake values were used in the statistical analysis, and geometric means and standard deviations are used to express average daily nutrient intakes. The percentage of energy from fat was fairly normally distributed so the traditional mean and standard deviation are reported and no transformation was used in the statistical analysis. For all the other food and beverage group variables (such as grains, fruits, milk, etc.), the data contained non-consumers (that is, students who reported eating 0 g of grains or fruits). Therefore, the geometric means and log transformation could not be performed. For those variables, the traditional mean and standard deviation are reported and a square-root transformation was used in the statistical analysis to help normalize the data.

General linear mixed models were used to analyze differences in means at the end of the study between intervention and control schools [17,18] with the covariance structure adjusting for variability both between cluster (school) and within cluster (students within the same school)[19,20]. Baseline values were included in the models as covariates. Analyses were repeated for those children with a BMI at or above the 95^th ^percentile at baseline (n = 1166). All analyses were conducted using SAS statistical software version 9.2 (SAS Institute, Cary, NC). P values < 0.05 were considered statistically significant.

## Results

Table 2 shows the baseline characteristics of the sample by intervention status. Randomization was effectively achieved with equal proportion of students by gender, race/ethnicity, parental educational status, family history of diabetes, and weight status in the intervention and control groups. These data also illustrate the high prevalence of overweight and obesity among sixth grade children across the country.

**Table 2:** Baseline Characteristics of the HEALTHY Student Cohort with Complete Dietary Data, N = 3908

Characteristic	Intervention (n = 1964)	Control (n = 1944)
Age in years	11.3 (0.5)	11.3 (0.6)

Gender male	47%	47%

Race/ethnicity		

Hispanic	56%	55%

Black	19%	14%

White	18%	22%

Other	7%	9%

Head of Household Educational Status		

Less than high school	12%	11%

Some high school	12%	14%

High school graduate	25%	26%

Some college or specialized training	31%	27%

College or univ grad	14%	15%

Postgrad training/degree	6%	7%

Family history of diabetes	19%	20%

BMI	22.3 (5.4)	22.2 (5.5)

BMI > = 85%ile	50%	49%

BMI > = 95%ile	29%	30%

Table 3 shows dietary intake estimated from the FFQ for the entire sample by intervention status at baseline and at end of study. There were significant differences between groups for mean intakes of fruit and water. The reported average daily fruit consumption, excluding 100% fruit juice, was 10% higher at the end of the study among children in the intervention schools than those in the control schools (138 g or approximately 2 servings versus 122 g, respectively, p = 0.0016). The reported water intake was approximately 2 fluid ounces higher among students in the intervention schools than those in the control schools (483 g versus 429 g respectively; p = 0.008). There were no significant differences between intervention and control students for mean intakes of energy or macronutrients at the end of the study, or in mean intakes of fiber, grains, vegetables, legumes, sweets, sweetened beverages, and higher- or lower-fat milk consumption.

**Table 3:** Baseline and End of Study Dietary Intake Measures for Intervention and Control Schools^1^

	Overall (N = 3908)			**Baseline BMI **≥ **95**^**th **^**Percentile (N = 1166)**		
	**Baseline**	**End of Study**	**P-value**	**Baseline**	**End of Study**	**P-value**

Energy (calories)^2^						

Control	1395 (1371, 886, 1022)	1410 (1391, 797, 955)		1325 (1294,826,963)	1295 (1260,706,836)	

Intervention	1363 (1308, 852, 1012)	1375 (1370, 797, 931)	0.406	1290 (1249,784,934)	1283 (1271,720,850)	0.922

Carbohydrates (gms)^2^						

Control	193 (190,127,145)	200 (200,119,139)		184 (180,116,141)	183 (183,105,123)	

Intervention	190 (183,124,145)	195 (196,119,137)	0.414	180 (170,116,137)	183 (186,108,124)	0.940

Protein (gms)^2^						

Control	47 (47,32,36)	46 (45,28,33)		45 (45,30,34)	43 (42,26,29)	

Intervention	45 (44, 30,33)	45 (45,28,31)	0.454	43 (41,28,31)	42 (42,26,30)	0.745

Fat (gms)^2^						

Control	49 (47,36,40)	48 (46,31,36)		46 (45,34,35)	44 (42,27,28)	

Intervention	48 (46,34,37)	47 (46,31,34)	0.523	45 (43,31,33)	43 (43,28,30)	0.864

Fat (% of calories)^3^						

Control	32 (32,6,7)	31 (31,6,7)		32 (32,6,7)	31 (31,6,8)	

Intervention	32 (32,6,7)	31 (31,6,7)	0.728	32 (32,6,7)	31 (31,6,8)	0.743

Fiber (gms)^2^						

Control	12 (12,10,11)	11 (11,9,9)		12 (11,10,11)	11 (11,8,8)	

Intervention	12 (11,9,10)	11 (11,9,9)	0.571	11 (11,9,10)	11 (11,8,9)	0.576

Grains (gms) ^3^						

Control	222 (185,146,172)	224 (193,137,163)		209 (173,143,169)	202 (175,124,145)	

Intervention	217 (181,142,160)	218 (186,137,162)	0.704	203 (172,134,149)	205 (170,128,151)	0.642

Fruits (gms) ^3^						

Control	160 (116,157,171)	122 (85,123,133)		167 (121,166,175)	126 (94,123,137)	

Intervention	155 (107,150,162)	138 (104,128,141)	0.002	155 (110,144,154)	136 (104,126,130)	0.059

Vegetables (gms) ^3^						

Control	87 (53,105,95)	66 (39,82,75)		87 (56,97,96)	67 (39,84,80)	

Intervention	80 (48,96,81)	66 (37,87,74)	0.895	77 (51,91,77)	64 (38,75,73)	0.853

Legumes (gms) ^3^						

Control	21 (0,43,27)	22 (0,46,27)		21 (0,41,27)	23 (0,49,27)	

Intervention	19 (0,38,25)	20 (0,45,23)	0.532	19 (0,38,25)	21 (0,45,25)	0.608

Sweets (gms) ^3^						

Control	58 (39,59,53)	57 (42,56,53)		50 (33,51,47)	45 (33,45,42)	

Intervention	55 (37,59,50)	55 (37,54,52)	0.349	46 (33,48,42)	43 (30,40,39)	0.734

Water intake (gms) ^3^						

Control	343 (203, 382, 677)	429 (237, 400,609)		395 (203,420,677)	531 (533,427,592)	

Intervention	366 (203, 398, 677)	483 (355, 420, 592)	0.008	400 (237,405,643)	531 (533,418,592)	0.889

Sweetened beverage intake (gms) ^3^						

Control	305 (185,351,299)	405 (250,431,448)		282 (177,325,265)	362 (212,409,389)	

Intervention	319 (185, 387, 302)	373 (248,379,421)	0.309	321 (177,391,310)	347 (240,361,355)	0.623

Fruit Juice intake (gms) ^3^						

Control	130 (71, 177, 151)	124 (67,169, 169)		121 (71,167,107)	123 (67,169,169)	

Intervention	129 (71, 194, 169)	124 (67,188,169)	0.782	128 (71,189,160)	125 (36,200,169)	0.467

2% fat and whole milk (gms) ^3^						

Control	107 (61,134,122)	111 (61,139,157)		93 (52,129,122)	102 (52,134,139)	

Intervention	101 (61,125,131)	109 (61,139,135)	0.616	96 (52,122,131)	97 (52,132,122)	0.495

1% fat milk (gms)^3^						

Control	27 (0,89,0)	29 (0,97,0)		33 (0,95,0)	42 (0,119,0)	

Intervention	26 (0,84,0)	26 (0,84,0)	0.956	26 (0,82,0)	27 (0,78,0)	0.252

^1 ^All the regression models used in the analysis were mixed models adjusting for the cluster effect of school and included with the baseline nutrient or food group value included in the model as a covariate.

^2 ^Geometric Mean (Median, Standard Deviation, Inter-quartile Range)

^3 ^Arithmetic Mean (Median, Standard Deviation, Inter-quartile Range)

Table 3 also shows the estimated dietary intakes of the subgroup of children who were obese (BMI ≥ 95^th ^percentile) at the beginning of sixth grade. In this subgroup, mean reported intakes did not differ significantly between students in the intervention and control schools at end of study, with the exception of fruits, which approached statistical significance (136 g versus 126 g respectively, p = 0.058).

A sensitivity analysis using more stringent cut points for defining outliers at the lower end of total energy (i.e., <800 or <1000 calories) found similar results as those presented above (data not shown).

## Discussion

The HEALTHY study was a five-semester, middle school-based intervention that integrated multiple components in nutrition, physical education, behavior change, and social marketing-based communications. Self-reported dietary intakes at the end of the study did not differ significantly between control and intervention groups, with the exception of small differences in fruit and water intake. In fact, the results show that intake of fruits and vegetables decreased in both intervention and control schools over the course of the study; a finding that may be related to the increased autonomy of teenagers and influence of peers on lifestyle behaviors[21]. Some [22-25] but not all [26-30] school-based interventions have reported improvements in dietary intake. A meta-analysis of seven school-based interventions reported that intervention students consumed on average 19% more of a serving of fruits and vegetables compared to control students[22]. Based on a standard fruit/vegetable serving of 70 g, this is comparable to our findings. Others have also found increases in water consumption but of a magnitude much greater than that shown here[31,32]. It is also possible that the significant findings for water and fruit intake may have been due to chance given the number of statistical tests conducted in the analysis.

The lack of a greater effect on dietary intake in this analysis may explain in part some of the HEALTHY study's main findings [5] and may have been attributable to several factors. These include but are not limited to: changing school food policies during the time frame of the study; the intervention may not have been long enough to influence or detect additional dietary effects; the intervention may not have penetrated deeply or widely into areas of the food environment outside of school that have been shown to influence student dietary intake, such as the home, the home neighborhood, the homes of peers, the school neighborhood, and food establishments frequented by students; and lastly the FFQ may not have captured dietary changes that did occur.

Changing school food policies that occurred during the intervention may have been a particularly important factor. Prior to the start of our intervention, the Child Nutrition and WIC Reauthorization Act of 2004 required that local education agencies address childhood obesity by developing school wellness policies[33]. The Institute of Medicine's report on childhood obesity was released a year later, in 2005, re-emphasizing the need for change in school food environments and practices[34]. As with any change in regulation at the federal level, there was a delay before schools began to comply[35]. The study's nutrition intervention, with goals and strategies in line with the regulation, began in the fall of 2006 while both control and intervention schools were developing and implementing wellness policies.

Another factor that may have weakened our overall effect on dietary intake is the varying length of time that schools required to implement the nutrition intervention goals. Some goals were easier to implement than others and some schools embraced change more quickly than others. For example, some schools readily permitted vending machines to be turned off, sweetened beverages to be replaced by water in vending machines and school menus to be modified, such as by increasing the number of fruits offered at meals. Changes such as these depended on who had authority over vending machine operations and menu development, which varied from school to school. Other changes required a year or longer to implement at most schools, such as bringing in new products that were lower in fat and higher in fiber. These changes also depended on modifying food procurement bids at the district level, which was typically done annually and with significant financial and logistical implications. In an analysis of data on foods and beverages from source documents maintained by school food service personnel, including work production sheets, food/beverage product specification sheets and/or labels, recipes, and menus it was possible to verify that some significant changes were made in the school meals programs in intervention schools by the end of the study. (Mobley CC, Stadler DD, Staten MA, El Ghormli L, Gillis B, Siega-Riz AM, Hartstein J, Virus A.: Effect of a Middle School-based Diabetes Prevention Intervention Program on Foods Selected by Students; the HEALTHY Experience, submitted) In the National School Lunch and a la carte venues, more intervention than control schools met all the nutrition goals, except for the goal of increasing fiber content and servings of fruits and vegetables. In the School Breakfast Program venue, more intervention than control schools lowered the fat content of foods and increased the amount of fiber grain-based foods/legumes served per student but did not differ in servings per student of these foods with >2 grams of fiber per serving. Intervention schools also eliminated >1% fat milk and served significantly less amounts of fat than control schools. However, intervention and control schools did not differ significantly in fiber, fruit, vegetables, or nonfat/1% fat milk amounts served per student. In a la carte venues, pizzas, donuts, and cakes were removed and lower nutrient-dense beverages were replaced by water in intervention schools. Thus, the duration of our intervention may have been too short, given the length of time required to make food purchasing and policy changes at the district level. Other school-based interventions such as Pathways have noted a similar limitation [36].

Although our intervention had multiple components that were well integrated, this particular combination of intervention strategies may have not been sufficient to penetrate into the other levels of the social-ecological model for levels of influence [37] which can improve overall dietary behaviors. There have been many secular changes in the last 30 years linked to increasing obesity rates[34]. These external influences have created an environment that is not always supportive of healthful eating. Overcoming these barriers will require interventions that penetrate many levels of the social-ecological model and go beyond the school setting. Targeting the home environment and food eaten away from home are very important for preventing obesity and while we used newsletters to inform parents of healthy eating and healthy activities this may not have been sufficient to change these environments[21].

The literature suggests that tools for measuring dietary intake, which are all presently self-reported, may limit our ability to assess change[38]. All methods of self-reported dietary assessment (e.g., 24 hour-recalls, FFQ, food records, and check lists) tend to underreport energy and nutrient intake[39]. In the HEALTHY study, we chose the FFQ because of its use in other similar populations, ease of implementation and relatively low cost, and we made the assumption that the measurement error inherent in this tool would be minimized since student intake was measured both at baseline and end of study. Thus, the findings presented here need to be interpreted within the context of this limitation.

In conclusion, the HEALTHY study, a five-semester middle school-based intervention that integrated multiple components in nutrition, physical education, behavior change, and social-marketing-based communications, resulted in significant changes to student's reported fruit and water intake. Subsequent interventions need to go beyond the school environment to overcome the many barriers that limit our ability to improve a child's overall dietary habits and subsequently weight status.

## Competing interests

The authors declare that they have no competing interests.

## Authors' contributions

AMSR wrote the analysis plan with input from other authors and drafted the manuscript, LE conducted the statistical analysis, and CM, BG, DS, JH, SV, AV and JB participated in the interpretation of the results and provided critical comments. All the authors were involved in the implementation of the study as well as read and approved the final manuscript.
